# Soil-Transmitted Helminth Infections and Correlated Risk Factors in Preschool and School-Aged Children in Rural Southwest China

**DOI:** 10.1371/journal.pone.0045939

**Published:** 2012-09-27

**Authors:** Xiaobing Wang, Linxiu Zhang, Renfu Luo, Guofei Wang, Yingdan Chen, Alexis Medina, Karen Eggleston, Scott Rozelle, D. Scott Smith

**Affiliations:** 1 Center for Chinese Agricultural Policy, Institute for Geographical Sciences and Natural Resources Research, Chinese Academy of Sciences, Beijing, China; 2 National Institute of Parasitic Diseases, Chinese Center for Disease Control and Prevention, Shanghai, China; 3 Freeman Spogli Institute, Stanford University, Stanford, California, United States of America; 4 Stanford University School of Medicine, Stanford, California, United States of America; 5 Department of Internal Medicine, Kaiser Permanente Medical Group, Redwood City, California, United States of America; Swiss Tropical and Public Health Institute, Switzerland

## Abstract

We conducted a survey of 1707 children in 141 impoverished rural areas of Guizhou and Sichuan Provinces in Southwest China. Kato-Katz smear testing of stool samples elucidated the prevalence of ascariasis, trichuriasis and hookworm infections in pre-school and school aged children. Demographic, hygiene, household and anthropometric data were collected to better understand risks for infection in this population. 21.2 percent of pre-school children and 22.9 percent of school aged children were infected with at least one of the three types of STH. In Guizhou, 33.9 percent of pre-school children were infected, as were 40.1 percent of school aged children. In Sichuan, these numbers were 9.7 percent and 6.6 percent, respectively. Number of siblings, maternal education, consumption of uncooked meat, consumption of unboiled water, and livestock ownership all correlated significantly with STH infection. Through decomposition analysis, we determined that these correlates made up 26.7 percent of the difference in STH infection between the two provinces. Multivariate analysis showed that STH infection is associated with significantly lower weight-for-age and height-for-age z-scores; moreover, older children infected with STHs lag further behind on the international growth scales than younger children.

## Introduction

Historically, controlling soil-transmitted helminth (STH) infections has been a challenge in China. Tremendous progress over the last 60 years has been made to control these parasites [1]. As recently as the 1960s many development experts applauded China’s health care system for its effective delivery of basic health services, including STH control, in rural populations [2]. Minimally-trained “barefoot doctors” lived in and visited remote villages, offering free treatment of common diseases and educating the population about disease prevention and healthy behaviors [3], [4]. These local health personnel often treated large numbers of children in schools, since schools have a concentration of the targeted population, making care programs accessible and inexpensive [5]. Treating STH infections was on their list of priorities [6].

In the 1980s, however, public funding for rural health declined precipitously [7]. The barefoot doctor system collapsed and rural residents were largely left to fend for themselves. It is only in the past several years that China has once again turned its attention to rural health. In the interim, many diseases that had been managed and better controlled appear to have re-emerged [1]. STH infections–perhaps due to their less clinically obvious and largely asymptomatic nature have re-emerged with marked increase in prevalence, especially in remote, impoverished and rural areas [8]–[11].

Over the last decade, high STH prevalence rates in various regions of China from Yunnan [8] to Fujian [9] to Hunan [10] have been observed. Nearly all of these studies have been small in size, typically limited to a single township or even a single village. The notable exception to this otherwise fragmented look at helminths across China is a large-scale national survey conducted by the Chinese Ministry of Health from 2001 to 2004 [11]. This survey included both urban and rural areas, and found national prevalence of STH infections to be 19.6%.

The aim of our study was to determine the prevalence and correlates of STH infections in over 1700 preschool and elementary school children in six rural and impoverished counties in Sichuan and Guizhou Provinces–defining the scope of the problem across a fairly large swath of rural China. Using multivariate regression and decomposition analyses, we identified: 1) correlates of infection that explain variance in the data, and 2) the independent association of the presence of STH infections with anthropometric measures, which are important markers of child development.

## Methods

### Study Design and Setting

A cross-sectional survey of impoverished children in rural Guizhou and Sichuan Provinces in Southwestern China was carried out in April and June of 2010. These two provinces were chosen because of their high rates of poverty and a humid climate that is conducive to STH infection. Three rural counties were randomly selected using computer-generated selection in each of these two provinces based on income level. The six counties were selected from the bottom quartile of the counties based on average net per capita incomes. The setting was defined as impoverished based on average net per capita incomes (2750 RMB/year in Guizhou and 4750 RMB/year in Sichuan), putting the individuals surveyed at the bottom quartile of China’s rural income distribution [12], [13].

### Study Population, Sample Size and Sampling Strategy

Children were studied with respect to socialization (before school and in school) and exposure to school environments. We identified two groups of children: a 3–5 year old group and an 8–10 year old group.

**Figure 1:**
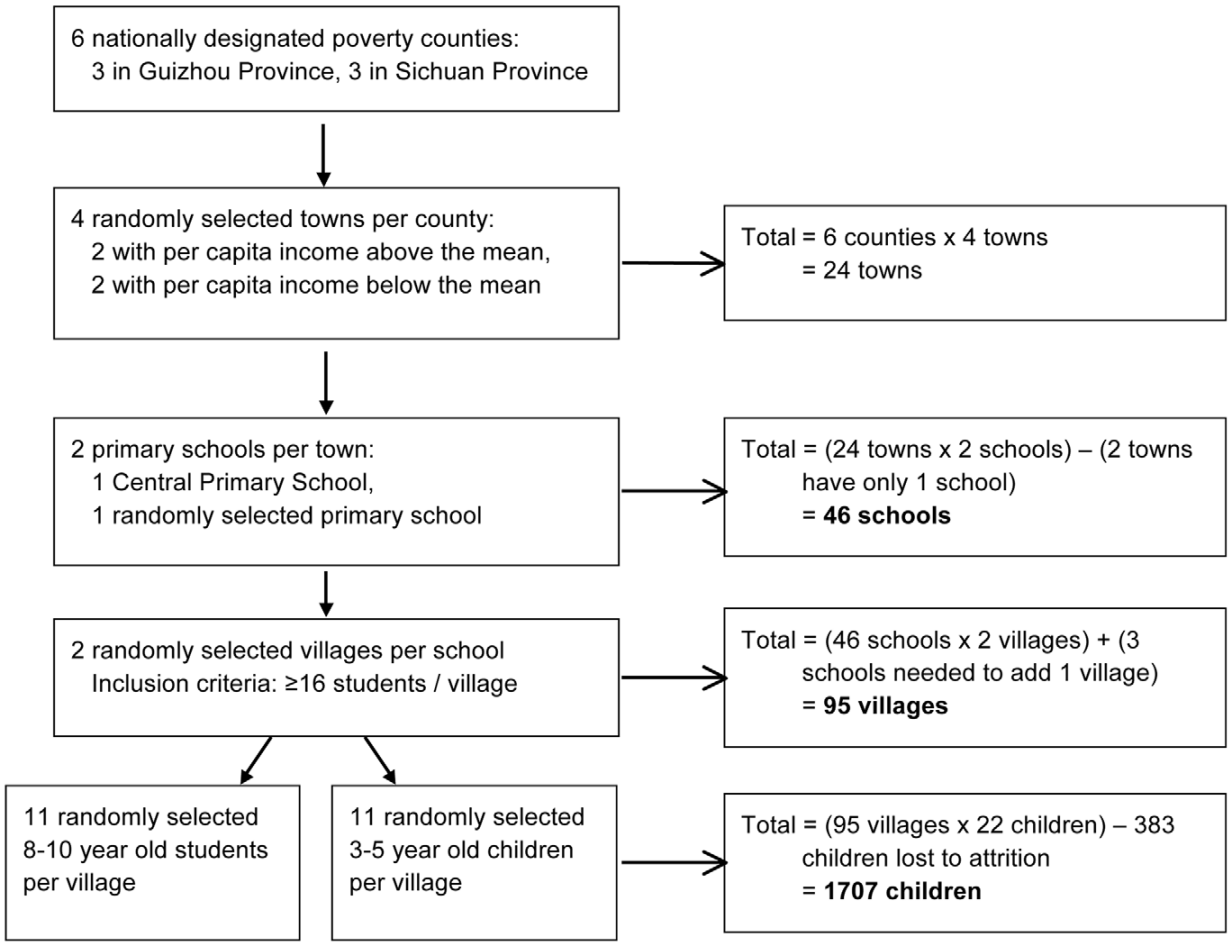
Sample selection schema.

A total of 141 areas, including 95 villages and 46 schools were studied in the survey. Details of the selection schema are shown in Figure 1.

In each of the six selected counties, we ranked all towns according to net income per capita, and then randomly chose four towns: two with income per capita above the mean for all towns in that county, and two with income per capita below the mean for all towns in that county. For each of the four chosen towns, two sample schools were chosen: the central primary school (which also serves as the local Bureau of Education’s administrative representative for all educational affairs in the town) and a randomly selected primary school. (Two of our sample towns had only one primary school, therefore the total number of sample schools is 46, rather than 48).

For each school, we obtained a list of all local villages that feed into the school. We ranked this list of villages by the number of 8–10 year old students (henceforth called ***school-aged*** children) enrolled at the school. We randomly selected two villages from the list (henceforth called ***sample villages***) that had 16 or more students enrolled at the school. We then randomly chose 11 enrolled students from each sample village. In each sample school, a total of 22 students were surveyed.

Next, we went to the sample villages to conduct the sampling of the 3–5 year old children. We obtained a list of all the 3–5 year old children in each sample village from the Registry of Child Immunization (which is recorded and stored in the town’s health center) and randomly chose 11 children from each of the sample villages (henceforth called ***preschool-aged children***).

In three of our sample schools, we were unable to find the requisite number of 3–5 year old children from the two corresponding sample villages. In those cases, we randomly selected a third village from the list of villages that feed into the school, and continued to select sample children from that village. This is why the total number of sample villages is 95, rather than 92 (46×2).

Our power calculations indicated that for our primary outcome variable, STH infection status, to estimate a 95% confidence interval with precision of 0.05 around a population prevalence of 40% and assuming an intra-cluster correlation (ICC) of 0.15, we required 8 children in each age group per village. We increased this to 11 children to account for attrition.

Overall then, in each sample village we randomly sampled 11 preschool-aged children, and 11 school-aged children. This led to sampling from a total of 817 pre-school aged children, and 890 school-aged children. Because some students refused to produce fecal samples, some sample villages had fewer than 22 observations with fecal samples. In no case were there fewer than 8 pre-school aged children and 8 school-aged children. On average, there were 9.75 school-aged children per sample village and 9.09 preschool-aged children per sample village.

### Data Collection and Survey

The primary outcome variable was STH infection status, which was determined by a single stool sample from each child participant. In addition, the intensity of every infection by egg density per gram was measured using WHO standard protocol [5]. Other variables and characteristics like household eating and sanitation information were collected on a socio-economic survey form. This survey contained data on each child’s age, gender, parental levels of education, health and sanitation behavior and other household characteristics. The survey also asked whether the child had taken anti-helminthic medication in the past 18 months. The school-aged children completed the survey themselves, in writing, under the direct supervision of trained enumerators from the Chinese Academy of Sciences. The preschool-aged children’s data was obtained by interviews with the parents, also by these trained enumerators.

Body height and weight were measured and recorded by trained nurses from Xi’an Jiaotong University according to WHO recommendations [14]. The children were measured in light clothing without shoes. Weight was measured with a calibrated electronic scale recommended by the medical department at Xi’an Jiaotong University. Body height was measured using a standard tape measure. The nursing team was trained to make sure the weighing station was set up on level ground to ensure accuracy of the equipment. Two nurses manned each measurement station, with one responsible for preparing subjects for measurement (removing shoes, offering instruction, reassuring parents, positioning children, etc.) and the other responsible for conducting and recording the actual measurements.

### Stool Sample Collection and Laboratory Testing

Stool samples of each of the children included in the study were collected once and sent to the local county Center for Disease Control & Prevention (CDC). There was one lab per county, or three labs per province, for a total of six labs. The majority of the samples were tested the same day that they were collected. Due to time and labor constraints, a small fraction of samples were tested the day after collection. These samples were stored overnight in the CDC laboratory refrigerator, which is kept at a constant 4°C. The Kato-Katz smear method was used for species specific identification of parasite eggs, including *Ascaris lumbricoides* (ascariasis), *Trichuris trichiuria* (whipworm) and hookworm. A single smear test was performed on each sample. Samples found to be positive for any of these three parasites underwent egg burden counts to determine eggs per gram (epg) of feces using standard WHO protocol [15].

CDC employees at the county level examined the samples and performed the tests. As a quality control, ten percent of samples were also checked by a parasite expert from the National Institute for Parasitic Disease to verify the initial diagnosis.

### Ethical Considerations

This study was approved by the Stanford University Institutional Review Board (IRB) on May 18^th^, 2010 and was assigned study protocol number 18780. The legal guardians (either parents or school principals) of all subjects provided informed oral consent, and the children themselves provided oral assent. The IRB approves the use of oral consents in rural China, to clarify understanding because many rural villagers are illiterate and it is culturally unusual to sign in writing. Our study enumerators recorded the consents on a list of names which is stored in a locked filing cabinet at the study center in Beijing, China.

Stool sampling falls within the regular purview of the Chinese Center for Disease Control & Prevention. They are professionally trained and perform routine stool sampling in the study areas as part of their national responsibilities. The stool sampling conducted as part of this study was approved and sanctioned by the national Chinese CDC as well as the local CDCs in the sample areas.

At the conclusion of the study, all participating children were treated with 200 mg of albendazole, per Chinese CDC national guidelines [16].

### Data Management and Statistical Analysis

To make sense of our data, we conduct both descriptive (univariate) and multivariate analyses.

As part of our univariate analysis, we grouped risks into four sets of factors: (1) deworming history; (2) individual characteristics; (3) eating and sanitation behaviors; and (4) household characteristics. Individual characteristics include age, gender, number of siblings and parental education. Eating and sanitation behaviors include handwashing after using the bathroom or before eating, the consumption of undercooked meat or vegetables, and the consumption of unboiled drinking water. Household characteristics include the type of toilet used by the household (either soil-based pits, including open defecation, or “other”, where “other” includes cement-lined pits or troughs, portable containers, and flush toilets); the material used to construct the floor of the home; the use of human fecal matter in household crop production; and whether the household raises livestock.

In order to better understand the strength of the correlations between each potential explanatory factor and infection prevalence, we conducted a multivariate regression analysis. Using a probit estimator (since our dependent variable is binary in nature–infected with STHs or not), we regressed STH treatment history; individual characteristics; health and sanitation behaviors; and household characteristics on STH infection, which we define as: “infection with any of the three types of STH.” The results of this analysis will help shed light on which factors are most strongly associated with STH infection.

Through our multivariate analysis, we will identify factors that influence STH infection. This information combined with any observed differences across survey regions can be used to identify factors that contribute to the variation in STH infection rates across different areas. This analysis, known as a decomposition analysis, uses a combination of the marginal impact of a variable on STH infection and the variation in that variable across regions to understand what share of the total variation in STH infection rate can be attributed to each variable.

## Results

### Prevalence of Intestinal Parasitic Infections

Overall, 21.2 percent of preschool-aged children and 22.9 percent of school-aged children were infected with one or more of the three types of STH tested for in the survey (Table 1). In Guizhou province, 33.9 percent of preschool-aged children and 40.1 percent of school-aged children tested positive for infection with one or more types of STH. In Sichuan province, only 9.7 percent of preschool-aged children and 6.6 percent of school-aged children were tested positive for infection.

**Table 1:** Prevalence of soil-transmitted helminth infections for two age cohorts in Guizhou and Sichuan, 2010.

	Preschool-aged children (Aged 3–5 years)			School-aged children (Aged 8–10 years)		
	Total(n = 815)	Guizhou(n = 386)	Sichuan(n = 429)	Total(n = 886)	Guizhou(n = 435)	Sichuan(n = 451)
Ascaris lumbricoides (%)	16.4^a^	29.5	4.7	18.6	32.2	2.4
Hookworm (%)	2.9	1.6	4.2	6.2	6.4	2.9
Trichuris trichiura (%)	4.9	6.7	3.3	9.7	14.3	2.2
**Any soil-transmitted helminth infection**	**21.2**	**33.9**	**9.8**	**26.1**	**39.8**	**6.7**
Prevalence of single infection	18.9	31.1	7.9	17.6	29.8	5.8
Prevalence of double infection	1.8	1.8	1.3	8.5	6.7	0.8
Prevalence of triple infection	0.7	1.0	0.5	0.0	3.2	0.0

a Totals may not sum exactly due to rounding.

**Figure 2:**
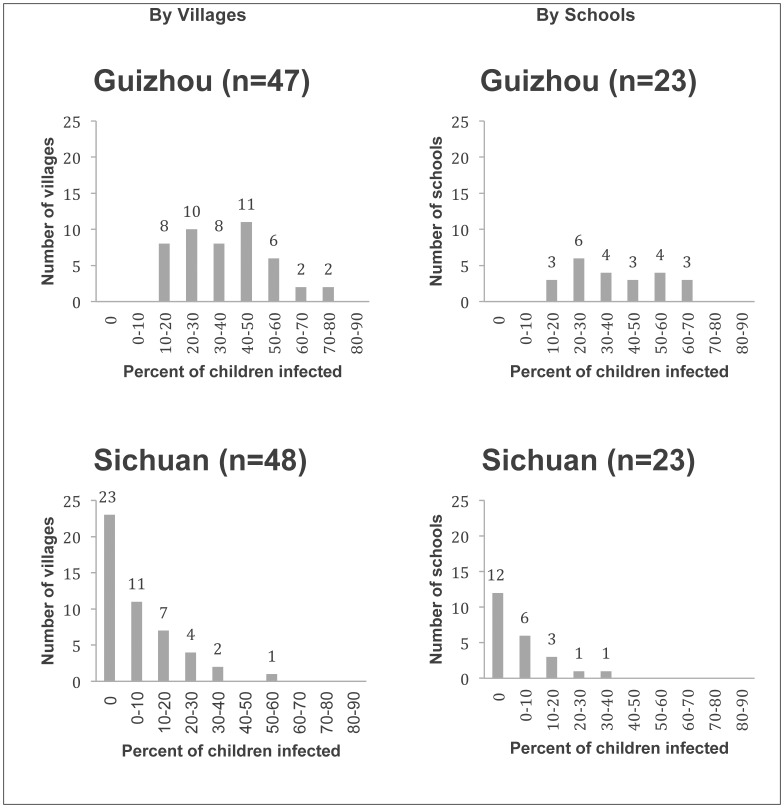
Numbers of villages and schools by prevalence of infection with A. lumbricoides, hookworm, T. trichiura, or any combination thereof in Guizhou and Sichuan, 2010.

Variation in prevalence across both villages and schools was observed in Sichuan (Figure 2). Seven of the 48 sample villages and two of the 23 sample schools have prevalence of 20 percent or higher. Twenty-five of the 48 sample villages and 11 of the 23 sample schools show STH infection rates greater than 0 percent. In Guizhou, ten of the 47 sample villages and seven of the 23 sample schools have infection rates of over 50 percent.

The results of the worm burden testing (eggs per gram of stool sample) are shown in Table 2. We find that for *A. lumbricoides*, 50.0% (n = 57) of preschool-aged children are characterized with having low intensity infections according to WHO guidelines, compared with 45.0% (n = 9) in Sichuan. Slightly higher percentages of school-aged children have low intensity *A. lumbricoides* infections in both provinces, at 52.9% (n = 74) in Guizhou and 81.8% (n = 9) in Sichuan. In both provinces, children with high intensity *A. lumbricoides* infections make up the smallest proportion of the total sample, at 13.2% (n = 15) and 10.7% (n = 15) among preschool-aged and school-aged children, respectively, in Guizhou, and 25.0% (n = 5) and 0.0% (n = 0) among children in Sichuan. Similar trends can be seen in the burden data for hookworm and *T. trichiura*: across both age groups and both provinces, the majority of sampled children have low intensity infections. The single exception to this rule is preschool-aged children infected with hookworm in Guizhou. Among this group, children with high intensity infections account for 66.6% (n = 4) of infected children, versus only 33.3% (n = 2) for children with low intensity infections; however, the sample size here is too small to allow the drawing of any meaningful conclusions.

**Table 2:** Eggs per gram in stool samples of infected children, by A. lumbricoides, hookworm and T. trichiura and by WHO category for two age cohorts in Sichuan and Guizhou, 2010.

	Preschool-aged children (Aged 3–5 years)		School-aged children (Aged 8–10 years)	
	Guizhou	Sichuan	Guizhou	Sichuan
**A. lumbricoides**				
Low (1–4,999)	57 (50.0%)	9 (45.0%)	74 (52.9%)	9 (81.8%)
Medium (5,000–49,999)	42 (36.8%)	6 (30.0%)	51 (36.4%)	2 (18.2%)
High (50,000+)	15 (13.2%)	5 (25.0%)	15(10.7%)	0 (0.0%)
Total	**114 (100%)**	**20 (100%)**	**140 (100%)**	**11 (100%)**
**Hookworm**				
Low (1–4,999)	2 (33.3%)	16 (88.9%)	20 (71.4%)	12 (92.3%)
Medium (5,000–49,999)	0 (0.0%)	0 (0.0%)	0 (0.0%)	0 (0.0%)
High (50,000+)	4 (66.6%)	2 (11.1%)	8 (28.6%)	1 (7.7%)
Total	**6 (100%)**	**18 (100%)**	**28 (100%)**	**13 (100%)**
**T. trichiura**				
Low (1–4,999)	22 (84.6%)	10 (71.4%)	52 (83.9%)	10 (100.0%)
Medium (5,000–49,999)	0 (0.0%)	2 (14.3%)	2 (3.2%)	0 (0.0%)
High (50,000+)	4 (15.4%)	2 (14.3%)	8 (12.9%)	0 (0.0%)
Total	**26 (100%)**	**14 (100%)**	**62 (100%)**	**10 (100%)**

### Univariate Analysis

We grouped risks into four sets of factors: (1) deworming history; (2) individual characteristics; (3) eating and sanitation behaviors; and (4) household characteristics (Table 3). 49 percent of the sample population in Guizhou and 46 percent in Sichuan took anti-helminth medication in the past 18 months.

**Table 3:** Descriptive statistics of independent variables used in the study, by province.

	Guizhou (n = 821)		Sichuan (n = 880)	
	Mean	95% ConfidenceInterval	Mean	95% ConfidenceInterval
Child has taken anti-helminth medicine in past 18 months (1 = yes, 0 = no)	50%	(46%–53%)	46%	(43%–49%)
**Individual characteristics**				
Gender (1 = male, 0 = female)	55%	(52%–59%)	46%	(43%–49%)
No. of family members (person)	5.1	(5.0–5.3)	5.2	(5.1–5.3)
No. of siblings (person)	1.2	(1.1–1.2)	0.9	(0.9–1.0)
Mother finished secondary school or above (1 = yes, 0 = no)	33%	(30%–36%)	56%	(53%–60%)
Father finished secondary school or above (1 = yes, 0 = no)	51%	(48%–55%)	64%	(61%–68%)
**Eating and sanitation habits**				
Wash hands before dinner (1 = yes, 0 = no)	67%	(64%–70%)	86%	(84%–89%)
Wash hands after using toilet (1 = yes, 0 = no)	59%	(56%–62%)	70%	(67%–73%)
Eat uncooked vegetables (1 = never, 0 otherwise)	25%	(22%–28%)	42%	(39%–45%)
Eat uncooked meat (1 = never, 0 otherwise)	80%	(78%–83%)	87%	(84%–89%)
Drink un-boiled water (1 = never, 0 otherwise)	19%	(16%–22%)	47%	(44%–50%)
**Household characteristics**				
Dirt floor (1 = yes, 0 = no)	17%	(15%–20%)	18%	(15%–20%)
Household has own toilet (1 = yes, 0 = no)	95%	(93%–96%)	96%	(95%–98%)
Soil-based latrine (1 = yes, 0 = no)	68%	(65%–71%)	79%	(76%–82%)
Use of human fecal material in household crop production (1 = yes, 0 = no)	77%	(74%–80%)	80%	(78%–83%)
Household owns livestock (1 = yes, 0 = no)	76%	(73%–79%)	69%	(66%–72%)
Use of human fecal material in household garden (1 = no, 0 = yes)	33%	(30%–36%)	30%	(27%–33%)

Individual characteristics included age, gender, number of siblings and parental education. In Guizhou, 45 percent of the sample is female and 55 percent is male; in Sichuan these numbers are 54 percent and 46 percent, respectively. Children in Guizhou have 1.18 siblings, on average, while children in Sichuan have 0.93. Only 33 percent of mothers in Guizhou finished secondary school, compared with 56 percent in Sichuan. For fathers, these numbers were 51 percent and 65 percent, respectively.

Eating and sanitation behaviors include handwashing after using the bathroom or before eating, the consumption of undercooked meat or vegetables, and the consumption of unboiled drinking water. In Sichuan, 86 percent of children reported washing their hands before dinner and 70 percent reported washing their hands after using the toilet. In Guizhou, these numbers were 67 percent and 59 percent, respectively. In Sichuan, 87 percent of children had never eaten uncooked meat, and 47 percent had never drunk unboiled water. In Guizhou, these numbers were 80 percent and 19 percent, respectively.

Household characteristics include the type of toilet used by the household (either soil-based pits, including open defecation, or “other”, where “other” includes cement-lined pits or troughs, portable containers, and flush toilets); the material used to construct the floor of the home; the use of human fecal matter in household crop production; and whether the household raises livestock. Households in Guizhou and Sichuan look fairly similar except for two factors. First, 68 percent of households in Guizhou use a soil-based latrine, versus 79 percent in Sichuan. Second, 76 percent of households in Guizhou raise livestock, versus 69 percent in Sichuan.

### Multivariate Analysis

Recall of whether a child has taken anti-helminth medication in the past 18 months has no significant effect on observed infection status (Table 4).

**Table 4:** Univariate and multivariate analyses of risk factors for STH infection (dependent variable) for sampled children in Guizhou and Sichuan, 2010.

Dependent Variable: STH infection (1 = yes, 0 = no)						
	Case	Controls	Univariate		Multivariate	
Variables	(n = 376)^a^	(n = 1325)^a^	Adjusted OR(95% CI)	*P*-value	Adjusted OR(95% CI)	*P*-value
Child has taken anti-helminth medicinein past 18 months (1 = yes, 0 = no)	164 (43.6)	647 (48.8)	0.81 (0.64–1.02)	0.07	0.85 (0.67–1.09)	0.20
**Individual characteristics**						
Gender (1 = male, 0 = female)	208 (55.3)	651 (49.1)	1.29 (1.02–1.62)	0.03	1.20 (0.94–1.52)	0.15
Age	6.9 (2.8)	6.7 (2.7)	1.03 (0.99–1.07)	0.15	0.99 (0.94–1.04)	0.63
Number of family members	5.2 (1.6)	5.1 (1.5)	1.04 (0.96–1.12)	0.34	0.99 (0.91–1.07)	0.72
Number of siblings	1.2 (0.9)	1.0 (0.9)	1.28 (1.13–1.45)	<0.001	1.18 (1.02–1.36)	0.03
Mother finished secondary school or above(1 = yes, 0 = no)	118 (31.4)	648 (48.9)	0.48 (0.37–0.61)	<0.001	0.63 (0.48–0.83)	<0.001
Father finished secondary school or above(1 = yes, 0 = no)	186 (49.5)	803 (60.6)	0.64 (0.51–0.80)	<0.001	0.90 (0.67–1.17)	0.44
**Eating and sanitation habits**						
Wash hands before dinner (1 = yes, 0 = no)	258 (68.6)	1053 (79.5)	0.56 (0.44–0.73)	<0.001	0.78 (0.58–1.05)	0.11
Wash hands after using toilet (1 = yes, 0 = no)	226 (60.1)	872 (65.8)	0.78 (0.62–0.99)	0.04	0.99 (0.74–1.33)	0.95
Eat uncooked vegetables (1 = never,0 otherwise)	114 (30.3)	465 (35.1)	0.80 (0.63–1.03)	0.09	1.05 (0.81–1.37)	0.72
Eat uncooked meat (1 = never, 0 otherwise)	296 (78.7)	1128 (85.1)	0.65 (0.49–0.87)	0.004	0.73 (0.53–1.00)	0.05
Drink un-boiled water (1 = never, 0 otherwise)	70 (18.6)	500 (37.7)	0.38 (0.29–0.50)	<0.001	0.48 (0.35–0.64)	<0.001
**Household characteristics**						
Dirt floor (1 = yes, 0 = no)	70 (18.6)	227 (17.1)	1.11 (0.83–1.50)	0.48	0.85 (0.62–1.18)	0.34
Household has own toilet (1 = yes, 0 = no)	358 (95.2)	1269 (95.8)	0.86 (0.50–1.49)	0.59	0.73 (0.41–1.30)	0.28
Soil-based latrine (1 = yes, 0 = no)	247 (65.7)	1004 (75.8)	0.61 (0.48–0.78)	<0.001	0.71 (0.54–0.93)	0.01
Use of human fecal material in householdcrop production (1 = yes, 0 = no)	304 (80.9)	1034 (78.0)	1.18 (0.89–1.58)	0.24	0.83 (0.59–1.18)	0.30
Household owns livestock (1 = yes, 0 = no)	306 (81.4)	926 (69.9)	1.88 (1.42–2.50)	<0.001	1.68 (1.19–2.36)	0.00
Use of human fecal material in householdgarden (1 = no, 0 = yes)	103 (27.4)	427 (32.2)	0.79 (0.62–1.02)	0.08	0.96 (0.73–1.27)	0.79

NOTE. ^a^Categorical data are no. (%) of subjects, continuous data are expressed as mean (SD)

OR = odds ratio; CI = confidence interval.

Mothers’ education is negatively correlates with children’s infection status. Specifically, the more educated the mother is, the less likely it is that her children are infected. With each additional year of maternal education, the probability of infection decreases by 7 percent (marginal effect is −0.07). In contrast, father’s education level has no impact on the probability of STH infection.

Health and sanitation behaviors were observed to correlate with STH infections. The consumption of undercooked meat is significantly and positively correlated with the probability of STH infection. Drinking unclean (unboiled) water is associated with a significant increase in the likelihood of infection.

Finally, soil-based latrine use is significantly and negatively correlated with infection, indicating that children living in households with soil-based latrines are *less* likely to have a child with STH infection. In addition, raising livestock is associated with a significant increase in the probability of STH infection of 8.0 percent.

### Decomposition Analysis

The difference in STH prevalence between Guizhou and Sichuan is 30.0 percent (Table 5). Table 5 shows that the statistically significant variables in Table 4 made up 24.14 percent of the difference in STH infection between the two provinces. Consistent with other studies [17], [18], 5.83 percent of the provincial difference in prevalence is due to differences in maternal education. The percentage of mothers with at least a secondary school education is 20 percent lower in Guizhou than in Sichuan. More siblings would have caused the infection rate in Sichuan to be 2.26 percent higher. The difference in infection rates between provinces derived from eating uncooked meat was 1.31 percent. Drinking unboiled water explains 10.69 percent of the difference. While the percentage of children who drink unboiled water was 81 percent in Guizhou, it was only 53 percent in Sichuan. Household characteristics explained 4.05 percent of the provincial difference in prevalence: soil-based latrines explained 2.18 percent, while owning livestock explained 1.87 percent.

**Table 5:** Decomposition analysis of the difference in STH infection rates between Guizhou and Sichuan, 2010.

Explanatory variables		Rate in Guizhou	Rate in Sichuan	Marginal Effect (on infection)	Explained infection rate	
		A	B	C	(A–B)*C	%
	% INFECTED	38%	8%		0.3	100
1	Number of siblings	1.18	0.93	0.026	−0.65	2.26
2	Percent with mother who finished secondaryschool or above	33	56	−0.073	−1.68	5.83
3	Percent who ever eat uncooked meat	20	13	−0.054	−0.38	1.31
4	Percent who ever drink un-boiled water	81	53	−0.110	−3.08	10.69
5	Percent with soil-based latrine	68	79	−0.057	−0.63	2.18
6	Percent whose household owns livestock	76	0.69	0.077	−0.54	1.87
	Explained infection rate (sum of rows 1–6)					**24.14**
	*Residual (all else)*					*75.86*

Overall, our data only explain 24.14 percent of the provincial difference in infection rates. This means that 75.86 percent of the difference is due to unexplained factors that we have not measured.

### Effect on Student Growth

Infection with one or more of the three STHs (*A. lumbricoides*, hookworm, or *T. trichiura*) is associated with significantly lower weight-for-age and height-for-age (Table 6). Moreover, there is a statistically significant negative coefficient on the age variable, indicating that older children infected with STHs lag further behind on the international growth scales than younger children.

**Table 6:** Ordinary Least Square (OLS) estimates of growth measures for sampled children in Guizhou and Sichuan, 2010.

Dependent variables	Weight-for-age z-score		Height-for-age z-score	
	OLS Coefficient (95% CI)	*P*-value	OLS Coefficient (95% CI)	*P*-value
Infected with any of three STHs(1 = yes, 0 = no)	−0.148 (−0.260–0.036)	0.009	−0.148 (−0.293–0.003)	0.046
**Individual characteristics**				
Gender (1 = male, 0 = female)	0.191 (0.101–0.282)	0.000	0.001 (−0.117–0.119)	0.987
Age	−0.024 (−0.043–0.005)	0.013	−0.041 (−0.066–0.016)	0.001
Number of family members	−0.019 (−0.050–0.013)	0.245	−0.013 (−0.054–0.028)	0.539
Number of siblings	−0.012 (−0.069–0.044)	0.665	0.028 (−0.045–0.102)	0.449
Mother finished secondary school or above(1 = yes, 0 = no)	0.146 (0.044–0.248)	0.005	0.097 (−0.036–0.231)	0.152
Father finished secondary school or above(1 = yes, 0 = no)	0.012 (−0.090–0.114)	0.816	0.097 (−0.035–0.230)	0.150
**Eating and sanitation habits**				
Wash hands before dinner (1 = yes, 0 = no)	0.193 (0.071–0.315)	0.002	0.195 (0.036–0.354)	0.017
Wash hands after using toilet (1 = yes, 0 = no)	−0.037 (−0.149–0.075)	0.519	0.079 (−0.067–0.225)	0.287
Eat uncooked vegetables (1 = never,0 otherwise)	0.019 (−0.079–0.117)	0.704	0.115 (−0.012–0.242)	0.076
Eat uncooked meat (1 = never, 0 otherwise)	0.051 (−0.077–0.179)	0.432	0.018 (−0.149–0.185)	0.832
Drink un-boiled water (1 = never, 0 otherwise)	0.140 (0.037–0.242)	0.008	−0.079 (−0.213–0.054)	0.242
**Household characteristics**				
Dirt floor (1 = yes, 0 = no)	0.042 (−0.082–0.167)	0.507	−0.107 (−0.269–0.056)	0.198
Household has own toilet (1 = yes, 0 = no)	−0.002 (−0.226–0.222)	0.987	−0.159 (−0.451–0.132)	0.284
Soil-based latrine (1 = yes, 0 = no)	0.128 (0.019–0.237)	0.021	0.049 (−0.093–0.190)	0.501
Use of human fecal material in household crop production (1 = yes, 0 = no)	−0.032 (−0.160–0.097)	0.629	−0.039 (−0.206–0.128)	0.644
Household owns livestock (1 = yes, 0 = no)	−0.085 (−0.204–0.034)	0.160	−0.161 (−0.317–0.006)	0.042
Use of human fecal material in householdgarden (1 = no, 0 = yes)	−0.079 (−0.179–0.022)	0.124	−0.023 (−0.154–0.108)	0.732
Constant	−0.615	0.001	−0.632	0.008
Adj-R^2^	0.051		0.024	
No. of obs.	1,699		1,697	

## Discussion

This study examines the prevalence and correlates of intestinal parasitic infection in children in impoverished rural regions of China where there is little published data or rigorous definition of the scope of the problem. We find that 33.9 percent of preschool-aged children and 40.1 percent of school-aged children in Guizhou tested positive for infection with one or more types of STH, while these numbers were 9.7 percent and 6.6 percent, respectively, in Sichuan. The national Ministry of Health survey of helminth prevalence from 2001–2004 included 356,629 individuals across China, and encompassed several studies by local Centers for Disease Control and Prevention (CDC), including in our study areas. The prevalence of *A. lumbricoides*, hookworm, and *T. trichiura* that we identify here support the results of these local CDC surveys, and indicate little change between 2002 and present-day. For example, Tang et al. (2005) found infection rates of 10.4%, 21.1%, and 2.6% for hookworm, *A. lumbricoides*, and *T. trichiura*, respectively, among 625 children under 5 years old in hilly regions of Sichuan province [19]. For children aged 5–9 years, these numbers were 11.0%, 23.0%, and 5.6%, respectively. In 2005, the Sichuan Provincial CDC reported an overall STH infection rate of 39.2%, among 6684 children under age 14 from 18 randomly selected counties [20]. The Guizhou Provincial CDC found STH prevalence of 53.8% among 4091 primary and middle school students, and prevalence of 48.3% among 1748 children of preschool age [21]. A more localized study in Guizhou found similar rates in 2007; specifically, the authors reported STH prevalence of 50.0% among children aged 2–12 years in a rural, mountainous county in western Guizhou [22].

We also identify factors that correlate with STH infection. Many of the correlates are not surprising, like the number of siblings, maternal education, consumption of uncooked meat, and consumption of unboiled water. These factors have been identified in other studies of STH infection elsewhere [23]–[25].

We found two unexpected results. Our first surprising result was the statistically insignificant effect of deworming treatment on STH infection. Why might this be? One possibility is that individuals who take deworming medicine may be taking an insufficient dose of the medication. National guidelines released by the Chinese CDC [25] recommend the annual administration of only 200 mg of albendazole for children aged 2–12 years, only half of the 400 mg dose recommended by the World Health Organization (WHO) [15]. Giving this lower dosage may have under-treated, thus leading to the ineffectiveness of the deworming treatment and probably sooner re-infection.

A second reason may be related to the frequency of treatment. The WHO treatment guidelines recommend that in communities with infection rates of 20% to 50% (the rate observed in our study areas), school-aged children should be treated once a year, since for the STHs considered in this study, reinfection occurs rapidly after treatment [26]. However, our survey asked individuals about their deworming history over the past 18 months, more than sufficient time for reinfection to have occurred.

In the context of the observed high rates of infection more frequent or structured and formalized population-level deworming efforts may be needed to better manage the observed infections. Currently, intestinal parasite control is left to the individual and there is no population-level regular intervention. Such programs have been successful in the past in China [6], and in other settings [17]. Health education and environmental sanitation programs have also been successful at reducing reinfection rates in other settings, and may be a prudent addition to China’s anti-helminth efforts [26].

Our second surprising result was that the use of soil-based latrines is negatively correlated with roundworm infection. Soil-based latrines are usually thought of as dirty environments conducive to STH survival and spread. However, it may be that soil-based latrines are actually *cleaner* than the alternative. In our sample villages, the two predominant types of latrine are soil-based and water-based. Soil-based latrines are, at their most basic, simply a large hole in the ground. Water-based latrines are large, lined troughs in the ground that are partially filled with water. The waste in water-based latrines is periodically drained to a nearby cesspool. Although soil leeching at the point of defecation is less likely in water latrines, the waste eventually covers a larger area, potentially offering more opportunities for contamination to occur. Few, if any, rural households have porcelain flush toilets that are commonplace in wealthier areas with plumbing.

Through our decomposition analysis we identify the correlating factors primarily responsible for the difference in prevalence between the two study areas. Unclean drinking water is the most important correlate, accounting for over 10 percent of the difference in prevalence between Guizhou and Sichuan.

Another important factor contributing to the difference in provincial infection rates is maternal education. Because mothers are often responsible for both food preparation and the health education of children in the family, they significantly influence the health of their children. If a child’s mother is educated, she is more likely to know about the dangers of STH infection and how to prevent it, and thus more likely to incorporate safe health behaviors into the home, including boiling water.

Our study also offers a quantitative look at the physical effects of STH infection. Children infected with STHs have significantly lower weight-for-age and height-for-age z-scores than do healthy children, putting them at risk for a number of conditions associated with undernutrition.

Unfortunately, the STH problem is receiving little attention from health officials, teachers or parents. Contrary to WHO guidelines, the decision to deworm in rural China is typically made at the individual level, rather than at the community level. The few deworming campaigns that exist appear to be ineffective, since deworming at the observed frequency appears to be uncorrelated with infection rates. The primary factors correlated with infection are low levels of maternal education and poor food preparation techniques such as drinking unclean water or eating undercooked meat. Accordingly, for intestinal roundworms to be effectively controlled, it appears that a two-pronged approach is needed: first, educating parents and students about how to prevent infection, and then ensuring that deworming programs offer anthelminthic medicines regularly so that children remain STH-free. This is consistent with the official recommendations of the World Health Organization [5].

### Limitations

Because only a single stool sample was collected on each child in this study, using the Kato-Katz preparation to determine infection status, there is likely a significant underestimation of infection. This underestimation is based on the fact that a single sample misses infection in an individual because of the temporal variation in egg excretion over hours and days. It has been shown that obtaining only a single sample for *A. lumbricoides* and *T. trichiura* can underestimate infection rates by up to 50% [18]. Similarly, in areas of lower endemicity for STHs, it is observed that a single smear sample may not be reliable in determining infection status, due to fewer shed eggs, again giving falsely low prevalence estimates in the population. Because only a single slide from the stool sample was prepared, this is a limitation in that it results in under-reporting of infection rates. It has been reported that sensitivity for low infection rates increases from 20 to 54% when going from a single sample to three fecal samples on separate days [27]. (The usual practice would be to obtain 3 specimens on separate days for immediate microscopic analysis.) Lastly, because the samples had to be obtained then transported to the central lab for each province, some time elapsed before their review and thus specifically in the case of hookworm, fewer cases were likely identified. This occurs because of the observed rapid desiccation of hookworm eggs in the stool samples, and has been shown to lead to severe underestimates of the density of this pathogen if samples cannot be examined immediately [28]. Thus because of single sampling and the time lapse between stool collection and expert review, there are likely significant underestimates of the actual rates and densities of intestinal parasitic infections in our population.

Another potential source of bias may have come from the administration of the socioeconomic survey forms. School-aged children answered questions about their individual, household, and health behavior characteristics on their own, while the same information was collected about preschool children by asking their parents. It is possible that this may have led to fundamental differences between the two age groups in the types of answers we received, although there is no reason to think that either group would systematically overestimate or underestimate in their responses. Regardless, any differences between the age groups should not affect our results since we control for the age of the child in our multivariate analyses.

### Conclusions

Our study shows an unexpectedly high prevalence of STH infections among children in impoverished areas of rural Guizhou and Sichuan provinces. There is considerable variation in prevalence rates between the two provinces that we sampled. We identified several factors that contribute to the difference in infection prevalence between Guizhou and Sichuan. Health and sanitation behaviors explain most of the explained difference; unclean drinking water and failing to wash hands before eating are among the most important correlates. Maternal education also plays an important role.

Student and parent recall about deworming treatments appear to indicate no significant correlation between deworming treatment and STH infection. This result hints at the ineffectiveness of sporadic deworming measures at an individual level, which allows for high rates of reinfection. It underscores the importance of a long-term, consistent deworming regimen.

Our study shows that STH infections still pose a significant health challenge to children in some poor, rural areas of China. Children infected with STHs have significantly lower weight-for-age and height-for-age z-scores than do non-infected children, putting them at risk for a number of conditions known to be associated with undernutrition.

We hope that this study will be the first of a series of studies that will start to define the scope of the problem, not only reporting the numbers observed, but also adverse outcomes such as the effects of infection on measures of nutritional status and school performance.

## References

[1] Wu G (2005) Medical Parasitology in China: A Historical Perspective. Chinese Medical Journal 118(9): 759–761. 15899139

[2] Wagstaff A, Lindelow M, Wang S, Zhang S (2009a) Reforming China’s Rural Health System. Washington, DC: The World Bank.

[3] Zhang D, Unschuld PU (2008) China’s Barefoot Doctor: Past, Present, and Future. Lancet 372(9653): 1865–1867. 18930539 10.1016/S0140-6736(08)61355-0

[4] Valentine V (2005) Health for the Masses: China’s ‘Barefoot Doctors’. National Public Radio, broadcast November 4.

[5] Montresor A, Crompton DWT, Gyorkos TW, Savioli L (2002) Helminth control in school-age children. Geneva: World Health Organization.

[6] Li T, He S, Zhao H, Zhao G, Zhu XQ (2010) Major trends in human parasitic diseases in China. Trends in Parasitology 26: 264–270. 20400374 10.1016/j.pt.2010.02.007

[7] Wagstaff A, Yip W, Lindelow M, Hsiao W (2009) China’s health system and its reform: A review of recent studies. Health Economics 18: S7–S23. 19551753 10.1002/hec.1518

[8] Steinmann P, Du ZW, Wang LB, Wang XZ, Jiang JY, et al (2008) Extensive multiparasitism in a village of Yunnan province, People’s Republic of China, revealed by a suite of diagnostic methods. Am. J. Trop. Med. Hyg. 78: 760–769. 18458311

[9] Xu L, Pan B, Lin J, Chen L, Yu S, et al (2000) Creating health-promoting schools in rural China: a project started from deworming. Health Promotion International 15: 197–206.

[10] Zhou H, Watanabe C, Ohtsuka R (2007) Impacts of dietary intake and helminth infection on diversity in growth among schoolchildren in rural south China: A four-year longitudinal study. American Journal of Human Biology 19: 96–106. 17160977 10.1002/ajhb.20588

[11] Coordinating Office of the National Survey on the Important Human Parasitic Diseases (2005) A National Survey on Current Status of the Important Parasitic Diseases in Human Population, China Journal of Parasitology and Parasitic Diseases. 23: 332–340. 16562464

[12] National Statistical Bureau of China (2009a) Guizhou Statistical Yearbook. China Statistical Press.

[13] National Statistical Bureau of China (2009b) Sichuan Statistical Yearbook. China Statistical Press.

[14] de Onis M, Onyango AW, Van den Broeck J, Chumlea WC, Martorell R (2004) Measurement and standardization protocols for anthropometry used in the construction of a new international growth reference. Food and Nutrition Bulletin 25: S27–S36. 15069917 10.1177/15648265040251S104

[15] World Health Organization (2006) Preventive chemotherapy in human helminthiasis: Coordinated use of anthelminthic drugs. Geneva: World Health Organization.

[16] Center for Disease Control & Prevention (2010) Treatment guidelines for soil-transmitted helminth infections. Official Disease Control Publication of the Administrative Office of the Ministry of Health 98. [in Chinese].

[17] Kasai T, Nakatani H, Takeuchi T, Crump A (2007) Research and control of parasitic diseases in Japan: current position and future perspectives. Trends in Parasitology 23: 230–235. 17350339 10.1016/j.pt.2007.02.011 PMC7106409

[18] Knopp S, Mgeni AF, Khamis IS, Steinmann P, Stothard JR, et al (2008) Diagnosis of soil-transmitted helminths in the era of preventive chemotherapy: Effect of multiple stool sampling and use of different diagnostic techniques. PLoS Neglected Tropical Diseases 2: e331. 18982057 10.1371/journal.pntd.0000331 PMC2570799

[19] Tang Z, Tian H, Yang W, Liu C, Zheng D, et al.. (2005) Investigation on Human Intestinal Helminthiasis in Hilly Areas of Sichuan Province from 2002 to 2003. Parasitoses and Infectious Diseases 3: 161–164. [in Chinese].

[20] Xie H, Zheng D, Yang W, Liu C, Tang Z, et al.. (2005) Parasitic Infections among Children in Sichuan Province. Parasitoses and Infectious Diseases 3: 181–183. [in Chinese].

[21] Wang S, Chen Z, Li A, Tang L, Xu L, et al.. (2008) Current status and analysis of important human parasitic diseases in Guizhou Province. Journal of Pathogen Biology 2: 450–453. [in Chinese].

[22] Chen X, Xia X (2010) Survey of intestinal nematode infection among children visiting a hospital in Puding County. Journal of Medical Pest Control 26. [in Chinese].

[23] Olsen A, Samuelsen H, Onyango-Ouma W (2001) A study of risk factors for intestinal helminth infections using epidemiological and anthropological approaches. J. Biosoc. Sci. 33: 569–584. 10.1017/s0021932001005697 11683225

[24] Norhayati M, Oothuman P, Fatmah MS (1998) Some risk factors of Ascaris and Trichuris infection in Malaysian aborigine (Orang Asli) children. Med J Malaysia 53: 401–7. 10971984

[25] Nyarango RM, Aloo PA, Kabiru EW, Nyanchongi BO (2008) The risk of pathogenic intestinal parasite infections in Kisii Municipality, Kenya. BMC Public Health 8: 237–242. 18620608 10.1186/1471-2458-8-237 PMC2478685

[26] Jia T-W, Melville S, Utzinger J, King CH, Zhou X-N (2012) Soil-transmitted helminth reinfection after drug treatment: A systematic review and meta-analysis. PLoS Negl Trop Dis 6(5): e1621. 22590656 10.1371/journal.pntd.0001621 PMC3348161

[27] Cartwright CP (1999) Utility of multiple-stool-specimen ova and parasite examinations in a high-prevalence setting. Journal of Clinical Microbiology 37(8): 2408–2411. 10405376 10.1128/jcm.37.8.2408-2411.1999 PMC85240

[28] Tarafder MR, Carabin H, Joseph L, Balolong E Jr, Olveda R, et al (2010) Estimating the sensitivity and specificity of Kato-Katz stool examination technique for detection of hookworms, Ascaris lumbricoides and Trichuris trichiura infections in humans in the absence of a ‘gold standard’. International Journal for Parasitology 40: 399–404. 19772859 10.1016/j.ijpara.2009.09.003 PMC2829363

